# Screening for probiotic properties and potential immunogenic effects of lactobacilli strains isolated from various food products

**DOI:** 10.3389/fmicb.2024.1430582

**Published:** 2024-10-29

**Authors:** Magdalena Kowalczyk, Joanna M. Radziwill-Bienkowska, Małgorzata A. Marć, Rafał Jastrząb, Jennifer Mytych, Paweł Siedlecki, Agnieszka K. Szczepankowska

**Affiliations:** ^1^Institute of Biochemistry and Biophysics, Polish Academy of Sciences, Warsaw, Poland; ^2^Research and Development Center, Olimp Laboratories, Dębica, Poland

**Keywords:** adhesion, gut persistence, immunomodulatory effect, lactobacilli, probiotics, proinflammatory cytokines, IL-8, IL-1β

## Abstract

**Introduction:**

Deceleration of disease progression and re-establishment of microbial balance in the gut is often achieved by application of lactobacilli strains. Their beneficial effects are associated with probiotic properties, which may be accompanied by immunomodulatory action at mucosal surfaces.

**Methods:**

To single out such strains, we screened almost three hundred lactobacilli isolates from eight genera and various food sources for acid and bile salt tolerance, adhesion to mucin as well as hemolytic activity and antibiotic susceptibility. Moreover, the immunomodulatory effects of cell-free supernatant (CFS) fractions of five lactobacilli strains were assessed using an *in vitro* cell line model.

**Results and discussion:**

By our rationalized selection approach, we identified thirty-five strains with probiotic potential and biosafety features. Additionally, we showed that CFS from the *Lactiplantibacillus* L_4 strain downregulates proinflammatory cytokines IL-8 and IL-1β. In contrast, IL-8 expression was found to increase after treatment with CFSs of *Lactiplantibacillus* L_2 and L_5 and IL-1β was upregulated by CFSs of *Lactiplantibacillus* L_1 and *Lactiplantibacillus* L_3. Overall, our result delineate a rational approach of selecting lactobacilli strains for probiotic development to support the gut microbiota equilibrium and reinforce the host immune system.

## 1 Introduction

Lactobacilli are a heterogeneous group of low G-C, gram+, non-pathogenic lactic acid bacteria (LAB) with a reputed “Generally Recognized as Safe” (GRAS) status (USA) and a “Qualified Presumption of Safety” (QPS) status (Europe), whose representatives can be found in different environments, such as milk, traditional (fermented) dairy products, plants and the gastrointestinal and urogenital tract of humans and animals. One of the most acknowledged lactobacilli probiotic strains is *Lacticaseibacillus rhamnosus* (Basonym: *Lactobacillus rhamnosus*) GG, for which a range of therapeutic effects have been demonstrated (Majamaa and Isolauri, 1997; Kalliomäki et al., 2003). Experimental and clinical data evidence positive health claims for other lactobacilli strains as well (Kechagia et al., 2013; Sazawal et al., 2006; Fijan, 2014). Together with bifidobacteria, lactobacilli constitute a group of microorganisms with the largest number of strains used as probiotics or “live microorganisms, which when administered in adequate amounts, confer a health benefit on the host” (FAO/WHO, 2002; Hill et al., 2014).

Characterization of bacterial isolates as potentially probiotic microorganisms is based on several general selection criteria which include tolerance to low pH and bile salts, and adherence to intestinal epithelial cells and/or mucins. Mucins are protein components of the mucus layer that covers and protects the underlying intestinal epithelium. Adhesion to mucins is considered one of the key factors contributing to the persistence of lactobacilli in the intestinal tract (Nishiyama et al., 2016). Thus, elucidation of the interactions between lactobacilli and mucin *in vitro* along with bile salts and low pH tolerance is regarded as a means of assessing survival strategies of LAB in the GI tract. Evaluation of probiotic traits may involve also additional properties, such as microbial growth stimulation in the presence of prebiotics, modulation of the host immune response, antimicrobial and antagonistic activity against potentially pathogenic microbes, antioxidant activity, or inhibitory effect against cancer cells; yet, not all selection criteria need to be fulfilled (Shokryazdan et al., 2017).

Among the ways probiotic strains, including lactobacilli, contribute in promoting prohealth benefits is the modulation of the host immune system. This most commonly occurs via interaction with gut mucosa and intestinal epithelium (Perdigón et al., 2002; Wells, 2011; Maldonado Galdeano et al., 2019). Immunomodulatory effects of lactobacilli can be elicited by whole live bacterial cells, heat-killed bacteria, cell components or post-culture cell-free supernatants (CFSs), which contain metabolites or bioactive molecules released to the medium (Rocchetti et al., 2023; Wan et al., 2018; De Marco et al., 2018). Bacteria (or their products) are recognized by the host through interaction with pattern recognition receptors (PRR), which activates a TLR-regulated signaling cascade leading to cytokine and chemokine production by the mucosal gut epithelium. Studies show that lactobacilli elicit a varying, strain-related effect on the host immune system that can impact both the innate and adaptive immune responses (Wells, 2011). The differences in immune reactions triggered by bacteria have been linked with microbial-associated molecular patterns (MAMPs), including surface molecules (proteins, carbohydrates, lipids), and with produced metabolites (Maslowski, 2019; Lee C. G. et al., 2023). Bacterial CFS-associated immune responses were reported to vary depending on bacterial species or strain as well as cell culture or animal model tested. Johari et al. (2021) showed that the supernatant fraction from *Limosilactobacillus reuteri* (Basonym: *Lactobacillus reuteri*) PTCC 1655 reduces the levels of proinflammatory cytokines TNF-*α*, IL-1 and IL-6 in differentiated human monocytic THP-1 cell line. In turn, the CFS of *L. rhamnosus* GG was found to increase the production of anti-inflammatory cytokines IL-4, IL-5, and IL-10 in the colon epithelial cell line HT-29 (Wan et al., 2018). Administration of CFS of *Lactobacillus acidophilus* DSM20079 resulted in increased IL-10 levels in plasma of piglets and an overall anti-inflammatory effect in peripheral blood mononuclear cells from Crohn patients and healthy individuals (Hidalgo-Cantabrana et al., 2020). Lactobacilli CFSs were reported also to exert immunostimulatory effects (Tao et al., 2006; Fujiya et al., 2007). CFSs from a mix of probiotic bacteria, including lactobacilli species, were shown to have an immune-boosting effect in RAW264.7 murine macrophages, including stimulation of TNF-*α* (Al-Najjar et al., 2024). The varying probiotic and immunomodulatory potential of bacterial strains and/or their CFSs showcase the need for detailed studies to develop customized, tailored products for prevention and treatment of specific diseases.

In our study, we evaluated almost three hundred lactobacilli strains isolated from different food products and of various genera for their tolerance to gastrointestinal tract (GIT) conditions, mucoadhesion, carbohydrate metabolic potential and the lack of *β*-hemolysis and antibiotic resistance. Results of our *in vitro* immunomodulatory tests using bacterial CFSs and epithelial cell line model point to a range of potential therapeutic applications that can be developed based on specific lactobacilli strains, including counteraction of inflammatory or allergic conditions.

## 2 Materials and methods

### 2.1 Bacterial strains and culture conditions

In the study, we used almost three hundred lactobacilli strains from IBB PAS Central Collection of Strains (COLIBB, Poland) isolated from non-commercial food products (i.e., sourdough, kefir grains, raw milk, sheep’s milk cheese “Oscypek”) and belonging to eight genera (Zheng et al., 2020) (Table 1). Strains were cultured on De Man, Rogosa and Sharpe (MRS) medium (Millipore) at 37°C in anaerobic conditions (Genbox anaer, bioMérieux), unless stated otherwise. Antibiotic susceptibility tests were performed on Iso-Sensitest broth (IST; Oxoid) with MRS broth (IST:MRS 9:1 ratio). Stock cultures were kept at −80°C in MRS broth containing 20% (v/v) glycerol. LAB strains (*Lactococcus lactis* IL1403, TIL448, IBB477) with previously evaluated adhesion levels were cultured on M17 medium (Oxoid) with 0.5% (w/v) glucose at 30°C in aerobic conditions and used as controls in adhesion tests (Chopin et al., 1984; Radziwill-Bienkowska et al., 2016; Le et al., 2013).

**Table 1:** Bacterial strains used in study.

Genus	Previous phylogenetic group	Source	Number of strains
*Companilactobacillus*	*Lactobacillus alimentarius* group	Sourdough	23
*Lacticaseibacillus*	*Lactobacillus casei* group	Raw milk	43
		Oscypek cheese	29
		Sourdough	1
*Lactiplantibacillus*	*Lactobacillus plantarum* group	Raw milk	42
		Sourdough	39
		Oscypek cheese	12
*Lactobacillus*	*Lactobacillus delbrueckii* group	Kefir grains	2
*Latilactobacillus*	*Lactobacillus sakei* group	Raw milk	1
		Sourdough	1
*Lentilactobacillus*	*Lactobacillus buchneri* group	Kefir grains	76
		Oscypek cheese	1
*Levilactobacillus*	*Lactobacillus brevis* group	Sourdough	27
*Loigolactobacillus*	*Lactobacillus coryniformis* group	Sourdough	2

### 2.2 Screening test of gastrointestinal survival properties

Resistance of bacterial strains to acid and bile salts, as well as their ability to adhere to mucus, were assessed based on the method described by Radziwill-Bienkowska et al. (2016) with some modifications for high throughput. For each tested strain, several freshly-grown colonies were taken and used to prepare 1-mL bacterial suspensions at OD_600nm_ 0.5 in three variants: (i) PBS pH 7.4 (control conditions), (ii) PBS pH 3 (acid stress conditions) and (iii) PBS pH 7.4 containing 3 g/L of bile salts (sodium cholate and sodium deoxycholate; Sigma-Aldrich) (bile salt stress conditions). After incubation for 1 h at 37°C, bacterial cells were centrifuged at 9,000 rpm for 3 min to remove the stress factor, resuspended in PBS pH 7.4, and enumerated using the drop plate method. The susceptibility to stress factors was expressed as a decline in orders of magnitude of bacterial counts in comparison to control conditions. Simultaneously, to test the mucoadhesive properties, bacterial suspensions in PBS pH 7.4 (OD_600nm_ 1) were incubated for 3 h at 37°C on 96-well polystyrene microtiter plates (ThermoFisher Scientific) coated with 10 mg/mL type III mucin from porcine stomach (Sigma-Aldrich). The unbound bacteria were washed away with water, and adherent bacteria were stained with crystal violet (Merck), as previously described (Radziwill-Bienkowska et al., 2016). Each microtiter plate included the control strains, as well as blank wells with PBS pH 7.4. The adhesion was expressed as the optical density (OD_583nm_) of stained cells. For each strain, the average value of three measurements was calculated.

### 2.3 Carbohydrate catabolism and growth stimulation by prebiotics

To determine sugar fermentation profiles of selected lactobacilli strains, API 50 CH system (bioMérieux) was used following the manufacturer’s protocol. The strips were read after 48 and 72 h of incubation at 37°C. The ability to grow on several prebiotics as sole carbon sources was verified as previously described by Dunislawska et al. (2017) with some modifications. Bacteria from freshly-grown colonies were suspended and diluted to OD_600nm_ 0.5 in MRS broth without dextrose (Pronadisa, Condalabs). Next, 5 μL of bacterial dilutions were inoculated in 200 μL of MRS broth without dextrose containing 0.5% of one of the following sugars: glucose (positive control), raffinose family oligosaccharides (RFOs) from lupin (Gulewicz et al., 2000), Bi2tos trans-galactooligosaccharide (B-GOS, Clasado Biosciences Ltd.), inulin from chicory (Sigma-Aldrich), inulin from dahlia tubers (Sigma-Aldrich), or no sugar (negative control), on honeycomb 100-well Bioscreen microplates. To obtain anaerobic conditions, a droplet of mineral oil (bioMérieux) was added on top to each well. The growth of bacteria was monitored at 37°C every 15 min for up to 70 h by measuring the OD_600nm_ with the Bioscreen C automated microbiology growth curve analysis system (Oy Growth Curves Ab Ltd). For some strains, OD_600nm_ values were determined by manual spectrophotometric measurements using Photometer LKT (Dr. Lange GmbH). Stimulation of bacterial growth by prebiotics was evaluated by comparing growth curves on medium containing prebiotics with medium containing glucose or no added sugar.

### 2.4 Safety assessment

The antibiotic susceptibility against nine antibiotics recommended for testing of bacterial strains intended for use as feed additives by EFSA (2012), namely ampicillin (AM), vancomycin (VA), gentamicin (GM), kanamycin (KM), streptomycin (SM), erythromycin (EM), clindamycin (CM), tetracycline (TC), and chloramphenicol (CL), was checked using Etest strips (bioMérieux) according to the manufacturer’s instructions. For some strains, minimal inhibitory concentrations (MICs) of KM (Sigma-Aldrich), EM (Sigma-Aldrich), CM (Sigma-Aldrich), and/or TC (BioShop) were confirmed using broth microdilution method according to the International Organization for Standardization (2010).^1^ *β*-hemolytic properties of lactobacilli strains were verified by streaking them on Columbia agar with 5% sheep blood (Becton, Dickinson & Co.) against *Streptococcus dysgalactiae* ssp. *equisimilis* H46A strain (IBB PAS collection) as a positive control. After incubation for 48 h at 37°C, plates were screened for the presence of hemolytic zones around the colonies. Strains with a clear halo around their colonies were considered as hemolytic (β-hemolysis), while non-hemolytic strains exhibited either a green zone (*α*-hemolysis) or no zone around the colonies (*γ*-hemolysis) (Zhang et al., 2022; Pradhan et al., 2020).

### 2.5 Preparation of bacterial cell-free supernatants

CFS samples were prepared from bacterial cultures grown on MRS broth at 37°C under anaerobic conditions (Genbag anaer; bioMérieux) to the early stationary phase. Next, 50 mL of culture was centrifuged (10,000 rpm, 10 min, 4°C), pellet was discarded and supernatant was filtered using a 0.2-μm membrane filter (VWR International) and concentrated 40 times using centrifugal concentrators with molecular cut-off of 50 kDa (Sartorius). Obtained CFS samples were neutralized (if necessary) to pH 7.0 by addition of 1 M NaOH and stored in aliquots at - 80°C.

### 2.6 Establishing the non-inhibitory CFSs concentration

Human colorectal adenocarcinoma cell line HT-29 (ATCC HTB-38™) was cultured in stable humidified conditions (95% air, 5% CO_2_) in McCoy’s 5A media with L-glutamine (Biowest), supplemented with 10% heat-inactivated foetal bovine serum (FBS) (EuroClone), 1% of non-essential amino acids (NEAA) (Gibco, Life Technologies) and penicillin and streptomycin antibiotics at 100 μg/mL final concentration each (Biowest) in cell culture flasks (25 cm^2^) to an appropriate density. Cells were then washed with modified Hank’s Balanced Salt Solution (Sigma-Aldrich), trypsinized using phenol-red Trypsin–EDTA (Sigma-Aldrich) and seeded on 96-well plates at 7,000 cells/well and cultured for 24 h. Next, cell culture medium was removed and freshly prepared dilutions (2–10%) of bacterial CFSs in McCoy’s 5A full culture media were added to each well in 100 μL aliquots and incubated for 24 h. Subsequently, the medium was removed and 100 μL of full fresh McCoy’s 5A medium was added into all the wells together with 20 μL of MTS reagent (CellTiter 96^®^ AQueous One Solution Cell Proliferation Assay; Promega) and incubated for 3.5 h. Cell viability was determined by measuring the absorbance on a microplate reader (Varioskan™ LUX multimode microplate reader; ThermoFisher Scientific) at 490 nm. All supernatants were tested in at least three independent biological experiments. Cell viability of untreated HT-29 cell culture served as a reference of growth conditions. IC_50_ values, signifying an inhibitory dose that reduces cell growth by 50%, were calculated according to the previously reported formula (Bacher et al., 2019).

### 2.7 Induction of cytokine expression in IL-1β-stimulated HT-29 cells

HT-29 cells were seeded on 6-well plates at 72,000 cells/well and cultured for 72 h, as described earlier. Subsequently, fresh bacterial CFSs were diluted in McCoy’s 5A complete medium containing IL-1β (0.001 μg/mL) and incubated with the cells for 24 h. The concentration of bacterial CFSs (and control bacterial MRS medium) was set at a non-inhibitory level of 2% (estimated experimentally on the basis of the MTS cell viability test). Then, prior to RNA extraction, all plates were controlled under the microscope, medium was removed and cells washed with Hank’s Balanced Salt buffer.

### 2.8 RNA extraction

Total RNA (from HT-29 cells cultured as a monolayer on 6-well plates) was extracted using standard RNAzol reagent (Sigma-Aldrich) according to manufacturer’s instructions. Concentrations and purity of the RNA samples were measured on μDrop Plate using the Varioskan Lux spectrophotometer (ThermoFisher Scientific) and diluted to a concentration of 0.4 μg/10 μL.

### 2.9 Reverse transcription and real-time PCR analysis

Total RNA was reverse-transcribed using High-Capacity cDNA Reverse Transcription Kit (Applied Biosystems, ThermoFisher Scientific) following the manufacturer’s protocol. Then, samples were diluted by adding 100 μL of ultrapure H_2_O to 20 μL of obtained cDNA. Quantitative RT-PCR (qRT-PCR) was performed on Azure Cielo 6 Real-Time PCR (Azure Biosystems) in 96-well plates with the use of TaqMan Universal PCR Master Mix (Applied Biosystems, ThermoFisher Scientific) and adequate amounts of TaqMan® Gene Expression Assay probes: *β*-actin, TNF-*α*, IL-6, IL-8 and IL-1β (Applied Biosystems, Life Technologies Corporation) according to the protocol supplied by the manufacturer. The assay was conducted under the following conditions: 10 min at 95°C, 70 amplification cycles comprising 15 s at 95°C, 1 min at 60°C. Samples were analysed using Azure Cielo manager and normalized to β-actin (control housekeeping gene) and reported according to the to the ΔΔCT method.

### 2.10 Statistical analysis

Presented data constitutes an average for independently performed experiments (three independent biological repetitions) with a standard deviation (SD). The GraphPad Prism™ 9 software (GraphPad Software) was used to calculate statistical significance, IC_50_ values of tested bacterial CFSs and data from qRT-PCR assays. Statistical significance was evaluated by one-way analysis of variance ANOVA, followed by Bonferroni’s post-hoc test (comparison between groups; statistical significance set at *p* < 0.05).

## 3 Results

### 3.1 Tolerance to gut conditions of lactobacilli strains

**Figure 1:**
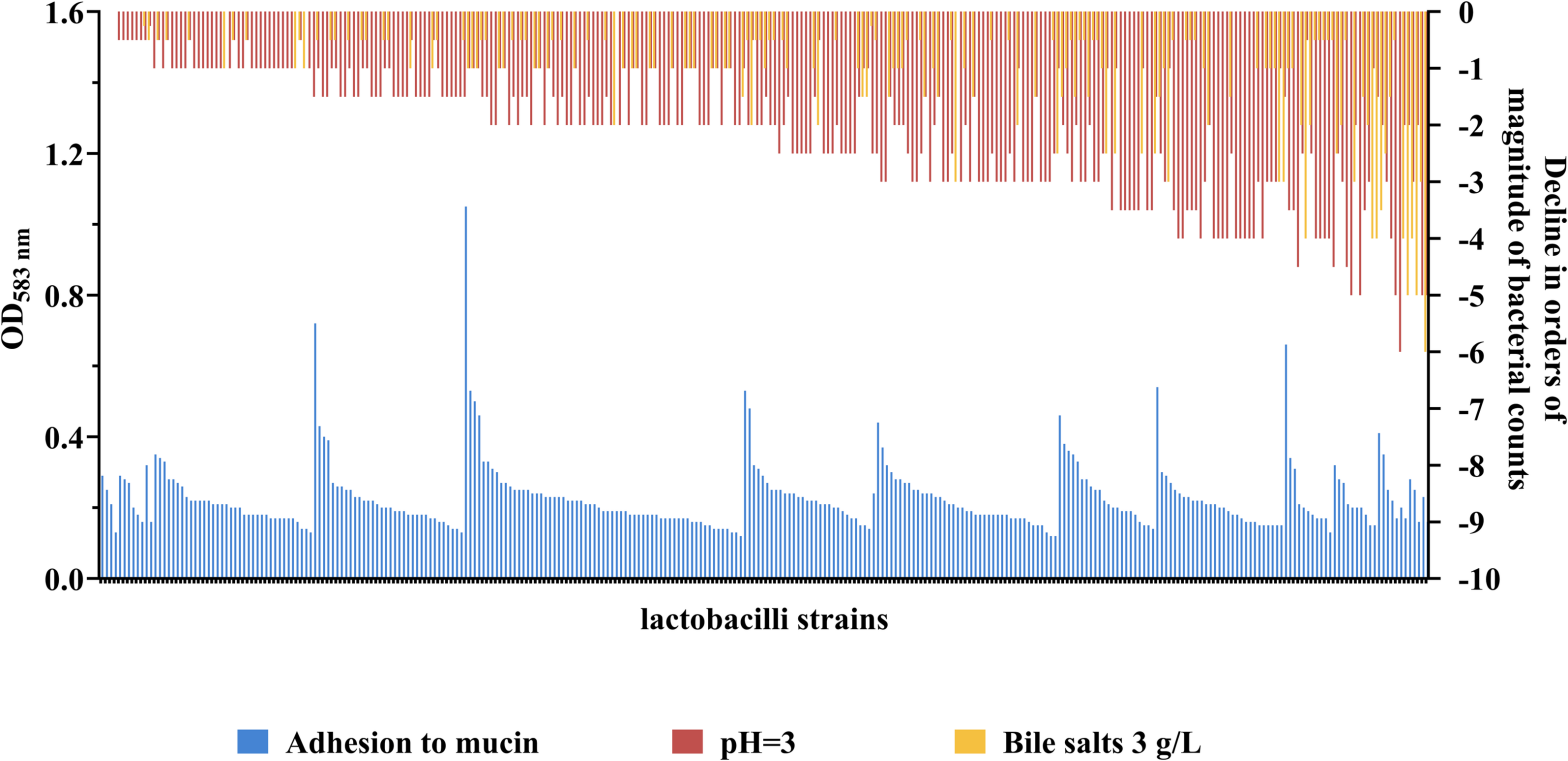
Strain-specific levels of adhesion and tolerance towards low pH and bile salts of lactobacilli. The level of adhesion to mucin is expressed in OD_583nm_ value - higher OD signifies better adhesion capacity. Control strains used to assess the level of adhesion: *L. lactis* TIL448 (strongly adherent strain; OD_583nm_ 0.54 ± 0.11), *L. lactis* IBB477 (semi-adherent strain; OD_583nm_ 0.27 ± 0.05) and *L. lactis* IL1403 (non-adherent strain; OD_583nm_ 0.18 ± 0.03). Sensitivity to bile salts (3 g/L) and low pH (pH 3) are expressed as the level of decrease in the viability of the tested strain in orders of magnitude relative to the viability of the same strain under control conditions (pH 7.4). Strains are ordered from lowest to highest sensitivity to bile salts and low pH (combined), and from highest to lowest adhesion within a given level of resistance to bile salts and pH.

To select for lactobacilli with probiotic potential, a rationalized experimental set-up was conducted on around three hundred strains of different origin from the IBB PAS Collection (Table 1). Strains were tested for their ability to survive in conditions imitating the GIT, including resistance to bile salts and low pH as well as the adherence to mucin (Figure 1 and Supplementary Figure S1).

**Figure 2:**
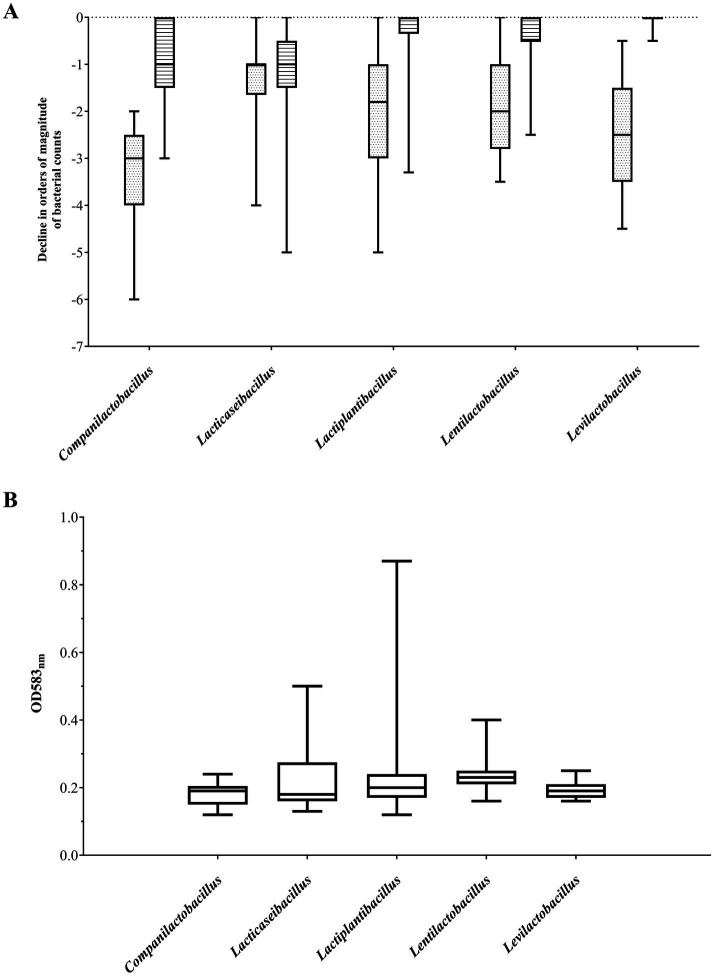
Resistance to bile salts and low pH (A) and adhesion to mucin (B) of the five most abundant lactobacilli genera. (A) Sensitivity to bile salts (3 g/L; striped boxes) and low pH (pH 3; dotted boxes) are expressed as the level of decrease in the viability of the tested strain in orders of magnitude relative to the viability of the same strain under control conditions (pH 7.4). (B) The level of adhesion to mucin is expressed in OD_583nm_ value - higher the OD signifies better adhesion capacity. Data is presented as a boxplot of obtained values (25th to 75th percentile with median line in the middle).

The most abundant group of bile salt tolerant strains were *Lactiplantibacillus* and *Levilactobacillus* isolates. The majority of strains from these two genera showed high resistance to its exposure, exhibiting no difference in viability compared to untreated bacterial cells (Figure 2A). Only less than 10% of the investigated *Lactiplantibacillus* strains weakly tolerated bile salts (above 1 log drop), while the viability of levilactobacilli did not fall below 0.5 log. In general, the most numerous group of bile-tolerant strains derived from sourdough and, among the tested *Lactiplantibacillus,* also from Oscypek cheese (Table 2 and Supplementary Figure S1). In turn, strains displaying the greatest sensitivity to bile salts were found in the *Lacticaseibacillus* group – around 20% of them exhibited a ≥ 2 log decrease in viability.

**Table 2:** Gastrointestinal viability screening of lactobacilli strains with different origin.

Genus	Source	No. of strains	Adhesion to mucin*	Bile salts sensitivity**	Sensitivity to low pH**
*Companilactobacillus*	Sourdough	23	0.12–0.24	0 to −3	−2 to −6
*Lacticaseibacillus*	Raw milk	43	0.14–0.50	0 to −5	−0.5 to −4
	Oscypek	29	0.13–0.27	0 to −3	0 to −1.5
	Cheese sourdough	1	0.28	−2	−5
*Lactiplantibacillus*	Raw milk	42	0.14–0.87	0 to −3.3	0 to −5
	Sourdough	39	0.12–0.41	0 to −0.8	−0.3 to −4
	Oscypek cheese	12	0.17–0.38	0 to −0.5	−0.3 to −2.5
*Lactobacillus*	Kefir	2	0.20; 0.25	−1; −3	−0.3; −4
*Latilactobacillus*	Raw milk	1	0.16	0	−4
	Sourdough	1	0.17	0.5	−5
*Lentilactobacillus*	Kefir	76	0.16–0.40	0 to −2.5	0 to −3.5
	Oscypek cheese	1	0.24	0	−2
*Levilactobacillus*	Sourdough	27	0.16–0.25	0 to − 0.5	−0.5 to −4.5
*Loigolactobacillus*	Sourdough	2	0.16–0.18	0	−4

*The level of adhesion to mucin is expressed in OD value at 583 nm; higher OD reflects stronger adhesion. Control strains used to assess the level of adhesion: *L. lactis* TIL448 (strongly adherent strain; OD_583nm_ 0.54 ± 0.11), *L. lactis* IBB477 (semi-adherent strain; OD_583nm_ 0.27 ± 0.05) and *L. lactis* IL1403 (non-adherent strain; OD_583nm_ 0.18 ± 0.03). **Sensitivity to bile salts (3 g/L) and low pH (pH 3) are expressed as the level of decline in viability of the tested strain in orders of magnitude (log drop) relative to the same strain not treated with a given agent. In bold are the lowest and highest values obtained for each genera.

At the same time, strains with relatively the highest resistance to low pH derived from *Lacticaseibacillus* spp. Half of representatives from this group demonstrated a 0–1 log drop in cell growth after exposure to pH 3 (Figure 2A). Among the analyzed *Lacticaseibacillus* isolates, the highest viability after incubation in acidic conditions presented strains deriving from Oscypek cheese. In contrast, the tested *Companilactobacillus* strains were the least tolerant to low pH; two-thirds of strains from this group showed a decrease in growth above 3 log (Table 2 and Supplementary Figure S1).

Lactobacilli strains under study exhibited differential potential to adhere to mucin. Considering five genera, for which more than twenty strains were tested (Table 1), non-adherent strains (below the level noted for the negative control strain) were observed among *Companilactobacillus* spp. and *Levilactobacillus* spp. (Figure 2B), while the majority of lentilactobacilli strains exhibited low adherence. The largest number of strongly adherent strains were noted among *Lacticaseibacillus* spp. (Figure 2B).

Taking into account the source of strain isolation (Table 2), most of our rye sourdough and kefir grains isolates were characterized by a relatively low level of adhesion and, at the same time, exhibited a high level of resistance to bile salts. The least sensitive to low pH were the investigated strains isolated from milk, which presented also the best adherence.

Overall, the conducted studies allowed to select for further assays forty strains, representing the most beneficial traits, being high bile salts and pH resistance, strong muco-adhesion, or a combination of all three properties.

### 3.2 Biosafety evaluation of lactobacilli strains

#### 3.2.1 Antibiotic susceptibility assays

The resistance of forty selected lactobacilli to nine antibiotic substances was examined using commercial Etests (Supplementary Figure S2). A strain was considered antibiotic-sensitive when the MIC value was not more than one dilution level higher than the break-point provided by the European Committee on Antimicrobial Susceptibility Testing (EUCAST). Based on the obtained data, none of the tested strains were found resistant to ampicillin, gentamicin, streptomycin or chloramphenicol. In turn, all lactobacilli strains showed resistance to vancomycin, which is in line with the intrinsic resistance to this antibiotic of most reported lactobacilli so far. Five *Lactiplantibacillus* strains and three *Lentilactobacillus* isolates displayed resistance to tetracycline exceeding the break-point MIC value. Kanamycin resistance was determined for four *Lacticaseibacillus* and one *Lactiplantibacillus* strain, whereas one *Levilactobacillus* and one *Lactiplantibacillus* were clindamycin resistant. For these strains, resistance to the three antibiotics (tetracycline, kanamycin and clindamycin) was tested by microdilution method. Based on the results from both assays, high resistance level was confirmed for two *Lentilactobacillus* strains to tetracycline, two *Lacticaseibacillus* isolates to kanamycin and one *Lactiplantibacillus* to clindamycin. As for these five strains the antibiotic resistance exceeded the cut-off MIC values, they were eliminated from further tests.

#### 3.2.2 Hemolytic activity test

An important biosafety feature of strains used as probiotics is lack of blood hemolytic capacity. All of the forty lactobacilli strains tested did not show any clear halos around the colonies on blood agar plates in comparison to the control *Streptococcus dysgalactiae* ssp. *equisimilis* H46A strain. For ten strains *α*-hemolysis was observed, while the remaining strains lacked any visible zones around the colonies (Supplementary Figure S2). Based on this observation, all strains were classified as non-*β*-hemolytic.

### 3.3 Growth stimulation of lactobacilli strains by prebiotics

Certain prebiotics can stimulate the growth of the probiotic strain, which may result in a beneficial impact on the host, including improvement of the gut barrier or fortification of the immune system. Thirty-five selected strains, which exhibited the most promising properties in gastrointestinal viability screening and displayed biosafety features, were examined for their ability to grow in medium containing prebiotics: RFO (Raffinose Family Oligosaccharides), B-GOS (Bi2tos trans-galactooligosaccharide) and two types of inulin—from dahlia and chicory, as a sugar source (Table 3 and Supplementary Figure S3).

**Table 3:** Sugar catabolism spectrum and growth promotion by prebiotics of tested lactobacilli.

Genus	No. of strains	Sugar catabolism spectrum*			Growth promotion by prebiotics***		
		Carbohydrate catabolism spectrum**	D-raffinose	Inulin	RFO	Inulin	B-GOS
*Lacticaseibacillus*	14	15-24	0	7	+(1)	++(5)	+(8)
							++(5)
						+++(2)	
							+++(1)
*Lactiplantibacillus*	16	19–24	10	1	–	–	+(5)
							++(10)
							+++(1)
*Lentilactobacillus*	3	6–21	1	0	+(1)	–	++(1)
							+++(1)
*Levilactobacillus*	2	7–8	0	0	–	–	+(1)

*The metabolic potential was assessed by API 50CH. **Values represent the number of metabolized sugars out of forty-nine tested. D-raffinose and inulin catabolism is presented as the number of strains capable of utilizing these compounds. *** Growth promotion by prebiotics was assessed by measuring cell density (OD_600nm_) in liquid culture; (−), no growth stimulation (growth as in standard medium without added sugar); (+), weaker growth than on standard medium with added sugar; (++), same growth as on standard medium with added sugar; (+++), better growth than on standard medium with added sugar. The number of strains meeting the given criteria is given in brackets.

The most beneficial effect on growth of the analyzed strains was shown for B-GOS, which: (i) influenced the growth dynamics better than the glucose medium (+++) for single strains from *Lacticaseibacillus*, *Lactiplantibacillus* and *Lentilactobacillus*; (ii) had the same effect as glucose medium (++) for sixteen strains from all groups, except *Levilactobacillus*; or (iii) had the same effect or slightly better than the pure medium without glucose (+) for eight *Lacticaseibacillus*, five *Lactiplantibacillus* and one *Levilactobacillus* strain, respectively. On inulin-containing medium, *Lacticaseibacillus* isolates showed the same (++; five strains) or better (+++; two strains) ability to grow than on medium with glucose. Notably, these were the only strains which growth was promoted in the presence of this prebiotic. Additionally, stimulation of bacterial growth by inulin occurred irrespectively to its origin (chicory or dahlia). RFO had the weakest impact on bacterial growth, affecting only two strains (one *Lacticaseibacillus* and one *Lentilactobacillus*).

### 3.4 Carbohydrate metabolic potential of lactobacilli strains

Establishing nutritional needs of probiotics is crucial for biotechnological manufacture as it can impact the growth and productivity of the strain. Thus, the thirty-five selected strains were assessed for their carbohydrate metabolic potential (Table 3 and Supplementary Figure S4). Overall, the widest range of carbohydrates was catabolized by the investigated *Lactiplantibacillus* and *Lacticaseibacillus* isolates. The ability to ferment D-tagatose was found only among strains from the *Lacticaseibacillus* group. The sourdough-originating *Levilactobacillus* spp. exhibited the lowest metabolic potential. Yet, strains from this group were able to degrade D-xylose which was a unique sugar metabolic trait among the tested lactobacilli. Inulin was degraded by bacteria from the *Lacticaseibacillus* group (plus a single *Lactiplantibacillus* strain). D-raffinose was metabolized by *Lactiplantibacillus* bacteria and one *Lentilactobacillus* strain (Table 3). Notably, there was no correlation between the strains’ carbohydrate metabolic potential to degrade D-raffinose or inulin (as determined by API 50 CH tests) and growth promotion by these prebiotics (Table 3).

### 3.5 Immunomodulatory effect of LAB-derived cell-free supernatants on epithelial cells

Probiotic bacteria during their growth release bioactive substances into the culture medium which can elicit immunomodulatory activity. To explore the potential of the secreted products on eukaryotic cells, we investigated cell-free supernatants (CFSs) of five out of the thirty-five strains presenting the most beneficial probiotic properties.

#### 3.5.1 Determination of non-inhibitory concentration of CFSs on cell viability

**Figure 3:**
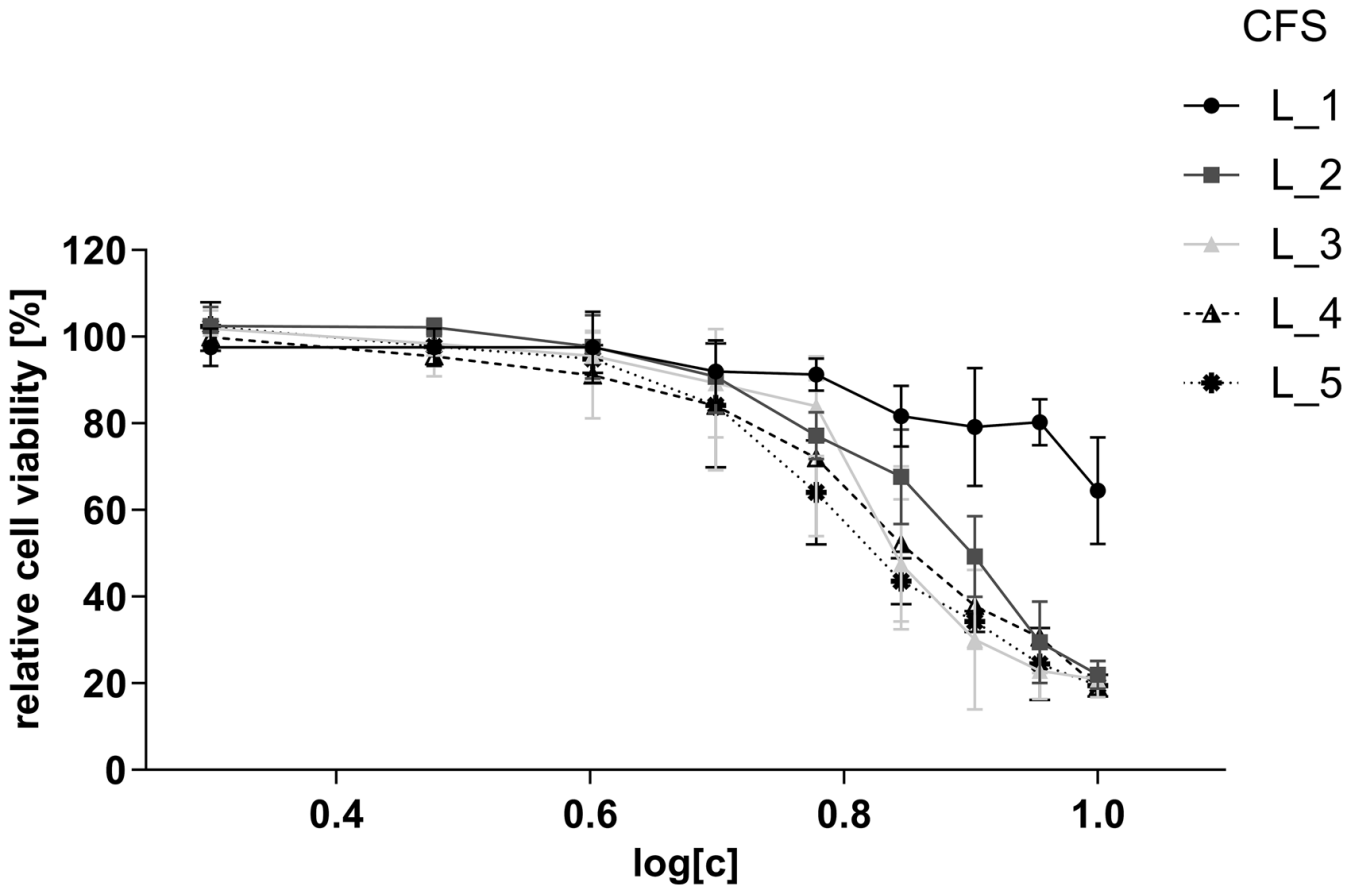
Viability of HT-29 cells after treatment with CFSs of lactobacilli strains. Relative cell viability ratio was calculated by dividing the absorbance obtained for CFS-treated HT-29 cells by the absorbance of untreated HT-29 cells (control) at equal concentrations. The assayed concentration ranges of CFSs are presented as log dilutions (2–10%). Data are presented as mean ± SD of at least three independent biological replicates.

To determine an appropriate concentration that would not compromise cell viability and could be applied in immunomodulatory assays, CFSs of five strains: one *Lacticaseibacillus* (L_1) and four *Lactiplantibacillus* (L_2, L_3, L_4, and L_5), were co-incubated at various concentrations with human intestinal epithelial HT-29 cells. Results of the MTS viability assay indicated that all CFSs, except one, had a comparable, strong inhibitory effect on cell viability (Figure 3). The calculated half-maximal inhibitory concentration (IC_50_) values for CFSs of L_2, L_3, L_4 and L_5 ranged between 4.28–6.23%, while for the L_1 supernatant the IC_50_ was 63.46% (Supplementary Figure S5). Based on these results, the dilution of CFSs for further assays was set at a non-inhibitory concentration level of 2%.

#### 3.5.2 Immunomodulatory effect of bacterial-derived supernatants in epithelial cells

**Figure 4:**
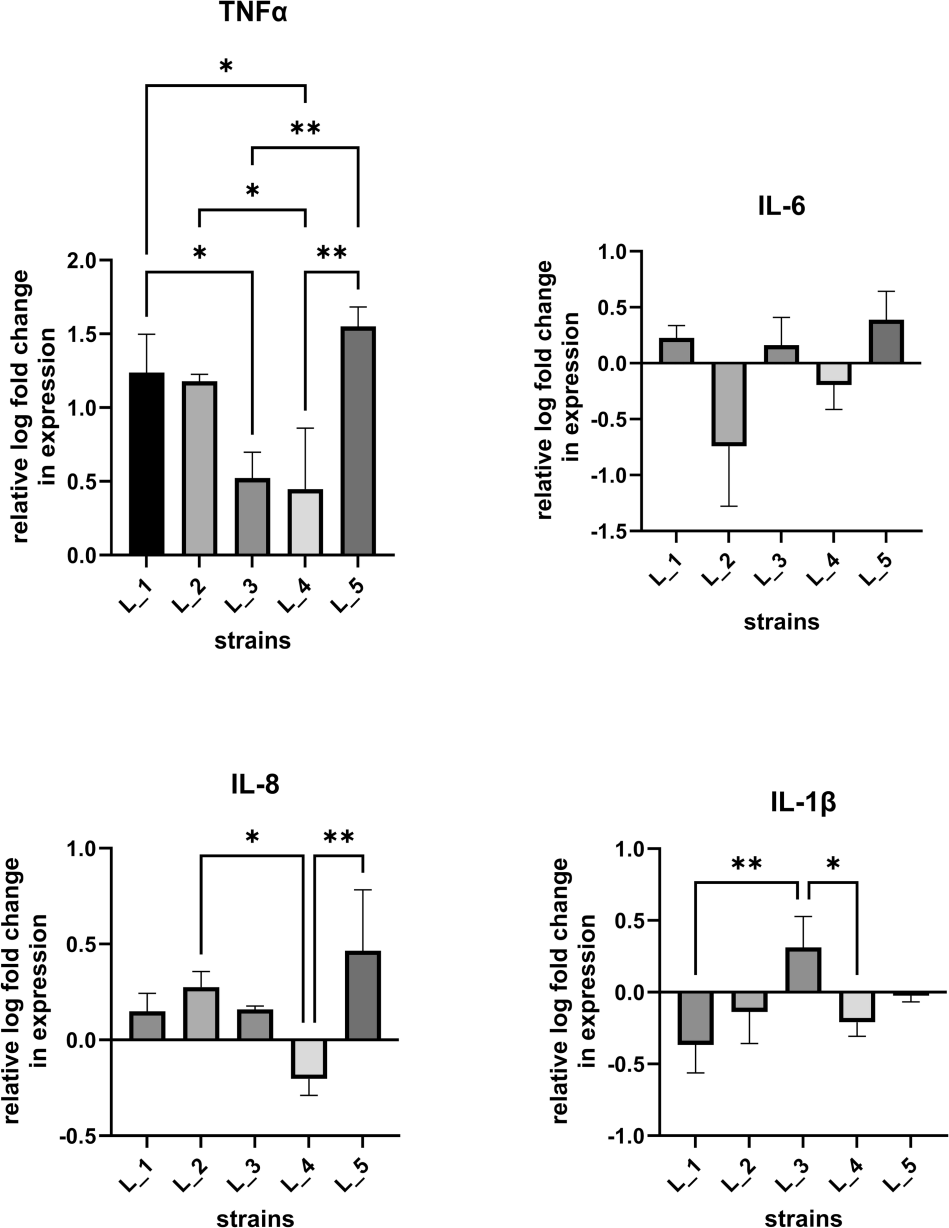
Cytokine expression levels after treatment of intestinal epithelial cells with lactobacilli CFSs. Real-time PCR was carried out to assess changes in IL-6, IL-8, IL-1β, TNF-*α* gene expression in IL-1β conditioned HT-29 cells after co-incubation with CFSs of lactobacilli strains. Data (*n* = 3, mean ± SD) are expressed as a relative log of mRNA levels in respect to cells treated with sterile culture medium (MRS). Statistical significance was evaluated by one-way analysis of variance ANOVA, followed by Bonferroni’s post-hoc test (*p < 0.05, ** *p* < 0.002).

To determine the influence of bacterial CFSs on cell immune responses, we investigated the proinflammatory cytokine expression (IL-1β, IL-6, IL-8, TNF-*α*) in IL-1β-stimulated human intestinal epithelial cell line (Figure 4; Supplementary Figure S6) as initial tests on naïve cells allowed only to observe baseline levels of induced cytokine responses (data not shown). HT-29 cells treated with CFSs of the five selected strains were found to induce a significant increase of TNF-⍺ levels. CFSs of two strains (L_2 and L_5) significantly upregulated and one (L_4) downregulated IL-8 expression. Statistically significant changes were also noted for L_3 CFS which slightly increased and for L_1 and L_4 CFSs which reduced IL-1β expression. Changes in IL-6 expression were also noted, but were statistically non-significant. Overall, the obtained data indicates a differentiating immune activation potential among the tested strains.

## 4 Discussion

Lactic acid bacteria (LAB) are a heterologous group of gram+ microorganisms, comprising species from *Lactococcus*, *Streptococcus*, *Enterococcus* genera, and the *Lactobacillaceae* family (Mokoena, 2017). Certain LAB strains carry the characteristics of probiotic strains. One of the most extensively studied groups of LAB are lactobacilli, which show prohealth potential in treatment of various illnesses, such as cancer, hypercholesterolemia, obesity or depression (Vivarelli et al., 2019; Zhang et al., 2020; Lakritz et al., 2014; Wang et al., 2019; Kang et al., 2023). They are part of the human commensal microbiota, and are also commonly found in raw milk, sourdoughs used for bread production or in traditional fermented milk products, such as Oscypek cheese and kefir (Rastogi and Singh, 2022; Arshad et al., 2018; De Vuyst et al., 2014; Kowalczyk et al., 2012; Alegría et al., 2012).

Desirable characteristics of probiotic strains intended for oral intake include high tolerance to low pH and bile salts, and high adhesion to gut epithelial cells and/or mucosa. To select the most prominent candidates for probiotic applications, we conducted an *in vitro* screening study of three hundred lactobacilli strains from various sources and taxonomic groups under conditions simulating gastrointestinal transit. Obtained data show that the examined traits are strain dependent. This suggests genetic variability among the individual isolates as previous works have argued that the beneficial health-promoting properties are linked with the presence of specific gene functions (e.g., bile salt hydrolases, proteins involved in response to stress or with mucoadhesive properties) (Salanski et al., 2022; Rebaza-Cardenas et al., 2023). Most tolerant to bile salts were the investigated *Lactiplantibacillus* and *Levilactobacillus* strains from Oscypek cheese and sourdough. Our *Lacticaseibacillus* spp. isolates from Oscypek cheese showed relatively the highest resistance to low pH. In turn, milk-isolated lactobacilli were the most adherent irrespective of the genera. These observations suggest that candidate strains possessing the respective genetic determinants may be frequently encountered among lactobacilli within these taxonomic groups and environments.

Characterization of the sugar catabolic spectrum of lactobacilli allowed us to determine the strains’ nutritional requirements and industrial potential. Most lactobacilli analyzed in this work catabolized a wide range of sugar sources, which indicated their ability to persist in niches with access to various carbohydrate substrates, including mono-, di- and polysaccharides. For certain lactobacilli, some unique sugar utilization patterns were observed (i.e., D-tagatose or D-xylose consumption). Degradation of D-xylose by levilactobacilli strains is indicative of their ability to utilize plant-derived carbon sources. Such property can be exploited in sustainable applications of lactobacilli for bioconversion of hemicellulose biomass into high-value products, such as lactic acid (Boguta et al., 2014; Tarraran and Mazzoli, 2018) and possibly also for starter cultures or adjunct probiotics cultures for plant-based food products. D-tagatose, as a sole sugar source, was shown to boost growth of intestinal lactobacilli – a property that can be considered in applications aimed at regaining microbial gut equilibrium over pathogenic bacteria (Bertelsen et al., 2001; Venema et al., 2005).

Bacteria utilize prebiotics in a strain-related manner, which is associated with the presence of specific gene clusters that encode saccharolytic enzymes degrading the prebiotic substrate. The knowledge on the consumption of specific prebiotics (e.g., galacto- or fructo- oligosaccharides) by probiotic strains was found valuable in selective enhancement of their propagation—a property that can be considered in applications aimed at fortifying the gut barrier, regaining microbial gut equilibrium over pathogenic bacteria, as well as in developing symbiotic (pre- and probiotic) formulations (Oliveira et al., 2011; Wilson and Whelan, 2017; Yadav et al., 2022). Growth of most lactobacilli in this study was stimulated by galacto-oligosaccharides (GOS), indicating that this is a preferable source of metabolized polysaccharides. GOS are the main component of milk in mammals and are known to shape the natural intestinal microbiota (Lee A. et al., 2023). In turn, only *Lacticaseibacillus* strains were able to metabolize and grow in the presence of a plant-derived fructan – inulin. The ability of tested lactobacilli to utilize for growth different prebiotics, i.e., GOS and inulin, suggests their adaptation to the milk and/or plant environment, respectively.

Aside the general positive impact probiotic strains have on host organisms, the specific mode by which they act may vary and is not fully understood (de Roock et al., 2010; Thomas and Versalovic, 2010; Bubnov et al., 2018). Among the recognized routes by which bacteria exert health-promoting effects is modulation of host immune cells via products secreted into the intestinal lumen (Cunningham et al., 2021). Use of bacterial secretions/CFS for therapeutic purposes is regarded as beneficial compared to probiotic formulations for certain groups of patients, such as immunodeficient or critically ill individuals and prematurely born neonates. Probiotic strains are considered to impact the host immune system via Th1- and Th2-mediated responses (Mazziotta et al., 2023). Promotion of Th1 response is associated with production of cellular proinflammatory immunity factors, such as interferon (IFN)-*γ*, interleukin (IL)-12, tumor necrosis factor (TNF)-*α* and IL-2, while the Th2-based response induces high levels of anti-inflammatory cytokine IL-10, IL-4 and transforming growth factor (TGF)-*β* (Ashraf et al., 2014). Our preliminary tests using human epithelial HT-29 cells showed that CFSs from five selected lactobacilli strains were able to trigger differential immune responses. For all investigated CFSs, we noted a strong upregulating effect on TNF-α expression. TNF-α is a proinflammatory cytokine of the Th1 response pathway engaged in activation of innate immunity and nonspecific immune reactions. The observed increase of TNF-α expression in IL-1β-sensitized cells after exposition to bacterial CFSs, suggests induction of Th1-mediated cellular immune mechanisms. Bacteria rendering such effect were proposed in several studies to have a therapeutic potential by acting as a countermeasure against the pro-allergic Th2 response and restoring the Th1/Th2 balance (Hougee et al., 2010; Eslami et al., 2020). Proinflammatory cytokines are key host immune components involved also in the activation of macrophages that play a crucial role in pathogen clearance and tumor suppression (Miettinen et al., 2000; Duque and Descoteaux, 2014). Probiotic strains and bacterial CFSs that promote generalized mucosal immune responses via proinflammatory cytokine production (e.g., TNF-α, IL-1β and IL-6) were found to fortify the host defense system and provide anti-pathogen and anti-tumor effects (Lee C. G. et al., 2023; Al-Najjar et al., 2024). In this line, the noted statistically significant upregulation of TNF-α and IL-1β by L_3 CFS and TNF-α and IL-8 by both, L_3 and L_5 CFS suggests their immune-boosting and anti-allergic potential.

In contrast, the CFS of *Lactiplantibacillus* L_4 downregulated the expression of IL-8 and IL-1β, and only weakly increased the TNF-α levels. Downregulation of IL-8 production was also observed in stimulated HT-29 cells using CFS derived from other lactobacilli strains, including CFS from *Lentilactobacillus kefir* IM002, *L. rhamnosus* R0011 and *Lacticaseibacillus helveticus* R0389 (Carey and Kostrzynska, 2013; Jeffrey et al., 2018). Such effects indicate induction of anti-inflammatory responses. Elevated IL-8 levels are associated with inflammatory processes observed during the onset of various illnesses, including human pulmonary diseases, cancer or autoimmune skin disorders, particularly atopic eczema (Pigossi et al., 2019; Naruke et al., 2021). Increased IL-8 levels have also been linked with the development of inflammatory bowel disease (IBD) (Mitsuyama et al., 1991; Daig et al., 1996). Luerce et al. (2014) demonstrated that the CFS of *L. lactis* NCDO 2118 strain can reduce the IL-8 levels in IL-1β-sensitized Caco-2 cells *in vitro* and ameliorate symptoms of chemically-induced colitis in mice. Hence, the ability to decrease IL-8 expression as manifested by L_4 CFS, could be especially valuable in developing treatments of inflammatory-based diseases, such as IBD.

## 5 Conclusion

Our comprehensive screening approach revealed unique individual properties of three hundred lactobacilli strains. We have shown that the probiotic and immunomodulatory potential of the tested bacteria are strain-specific. The differential tolerance to GIT transit and effects on the gut epithelial cells may be connected with variations in specific gene content and metabolites produced by these bacteria. The fact that the investigated lactobacilli derived from dairy products and in large part exhibited biosafety properties allows to consider them useful in food-based applications. Detailed further examination of strains exhibiting the most beneficial traits and their supernatant fractions, which evoke host immune responses, is highly warranted for developing novel pro- and postbiotic-based strategies for targeted therapies.

## Data Availability

The original contributions presented in the study are included in the article/Supplementary material, further inquiries can be directed to the corresponding author.

## Footnotes

^1^ https://www.iso.org/standard/46434.html

## Supplementary material

SUPPLEMENTARY FIGURE S1 Screening results of 300 lactobacilli strains for low pH and bile salt resistance and adhesion abilities.

SUPPLEMENTARY FIGURE S2 Biosafety assessment data for lactobacilli strains.

SUPPLEMENTARY FIGURE S3 Data on sugar catabolic potential of lactobacilli strains.

SUPPLEMENTARY FIGURE S4 Growth test results in the presence of prebiotics.

SUPPLEMENTARY FIGURE S5 MTS results after treatment of HT-29 cell with cell-free supernatants of five lactobacilli strains and cell viability curves.

SUPPLEMENTARY FIGURE S6 qRT-PCR data.
